# Elevated serum triglyceride levels may be a key independent predicting factor for gallbladder cancer risk in gallbladder stone disease patients: a case–control study

**DOI:** 10.1186/s12902-022-01189-y

**Published:** 2022-11-08

**Authors:** Yong Wan, Jianqin Zhang, Min Chen, Mao Ma, Binwu Sheng

**Affiliations:** 1 Department of Geriatric Surgery, First Affiliated Hospital of Xi’an Jiaotong University, No 277 West Yanta Road, Xi’an, China; 2 Shaanxi Nutrition Society, Medical School, of Xi’an Jiaotong University Xi’an, Shaanxi, China; 3 Biobank, First Affiliated Hospital of Xi’an Jiaotong University, Xi’an, Shaanxi China

**Keywords:** Gallbladder cancer, Gallbladder stone diseases, Hypertriglyceridemia, Insulin resistance, Metabolic syndrome

## Abstract

**Background:**

Gallbladder stone diseases (GSD) is a main risk factor of gallbladder cancer (GBC). This study aimed to reveal their bridge to metabolic syndrome.

**Material/method:**

The clinical and experimental data of 2210 GBC patients, from 3524 Chinese patients, in our hospital from Jan. 2009 to Dec. 2020 were summarized. The metabolic syndrome indexes, influencing factors for both GBC and GSD, were analyzed by unconditional logistic regression in this case–control study.

**Result:**

There were significantly higher morbidity of GBC in the overall, GSD and non-GSD with hypertriglyceridemia patients versus non-hypertriglyceridemia ones (*P* < 0.001, all). In GSD patients, univariate regression showed a significantly positive correlation between serum triglyceride (TG), low density lipoprotein cholesterol (LDL-c), fasting insulin (FINS) levels, Homeostasis model assessment-insulin resistance (HOMA-IR), female being, body mass index, hypertriglyceridemia and hazard of GBC with GSD (*P* < 0.001, all), and a significantly negative correlation to systolic pressure (SBP), diastolic pressure (DBP), hypertension and high-density lipoprotein cholesterol (HDL-c), fasting blood glucose (FBG) (*P* < 0.05, all); multivariate regression showed that serum triglyceride was the most significantly positive factor associated to GBC (*P* < 0.001, all) among the hazard factors including serum TG, LDL-c levels, HOMA-IR. In non-GSD ones, multivariate regression showed that HOMA-IR was the most significantly positive factor associated to GBC among the hazard factors including serum TG, LDL-c levels, HOMA-IR, female being, while DM had a significantly inversion negative association (*P* < 0.001).

**Conclusion:**

We found initially that elevated serum TG levels could be the most remarkable independent predicting factor for GBC risk with GSD, while insulin resistance might act as the first one in non-GSD. More importantly, we advocated initially the sharp rise of serum TG levels as the potential of a candidate diagnostic or prognostic biomarker of GBC with GSD.

**Trial registration:**

The study may be performed in accordance with the ethical standards provided by the responsible committee of our institution (First Affiliated Hospital of Xi’an Jiaotong University. XJTU1AF2020LSK-160) at which the work was carried out an in accordance with the Declaration of Helsinki. The ethics committee of our institution strictly comply with the requirements of ICH-GCP、GCP and relevant regulations to construct, operate and implement operating procedures.

## Background

Gallbladder cancer, highest burden in China, is the fifth most common cancer involving the gastrointestinal tract, and its five-year survival rate is only 5%; but it is the most common malignant tumor of the biliary tract worldwide [1, 2]. Its main risk factor is prolonged exposure to gallbladder stone disease (GSD), a common gastrointestinal disease, which is prone to develop into severe cholecystitis, or even strongly associated with gallbladder, pancreatic and colorectal cancer occurrence, and the National Institutes of Health estimated that almost 3,000 deaths (0.12% of all deaths) per year were attributed to the complications of GSD [3]. The association might result from their common initial etiologic including bacterial infections and other inflammatory conditions [4]. Singh, T. D. et al. studied the promoter methylation of certain tumor associated genes in the molecular pathogenesis of GBC and GSD and revealed that downregulation of SOCS1 only occurred in GBC and methylation frequency increase of 14–3-3 sigma, MASPIN and THBS1 genes from early to advanced GBC grades; with the latter several genes showed in GSD significantly as well [5]. There exists a strong link between the burden of GSD, especially for cholesterol gallstones, and highly prevalent metabolic disorders such as obesity, dyslipidemia, type 2 diabetes, hyperinsulinemia, hypertriglyceridemia and the metabolic syndrome [6, 7], as well as GBC [8, 9].

Although a personal history of gallstones has long been found to be strongly related to GBC risk, aetiology of GBC remains poorly understood. Cholecystectomy (or some other form of gallstone therapy) is indicated in most patients with symptomatic cholelithiasis – especially those with non-functioning gallbladders. But the issues of its complications and the optimal surgical time should not be ignored [10, 11]. Besides, it might be the best choice to cut off the links between GSD and GBC. Of the above, lifestyle changes including weight loss, more exercise, prevention and treatment of hyperlipidemia and diabetes mellitus will be the most possible to be realize. People in northwestern China feed on foods with high lipid and sugar, and with the westernization of diet in recent years, epidemiological investigations reported their stronger inclination to develop metabolic syndrome such as hyperlipidemia, obesity, diabetes mellitus and non-alcoholic fatty liver disease (NAFLD). Therefore, this study recruited subjects from in this typical area, and reviewed the clinical records of patients with GBC during a 12-year period, so as to reveal the bridge relating to metabolic syndrome between GBC and GSD. It may shed light the research of the risk factors of GBC development and provide preventive approaches for further gallbladder cancer resulting from GSD.

## Methods

### Study population

Anthropometric and laboratory parameters of 3524 Chinese GBC patients, were retrospectively reviewed at the time of preliminary diagnosis before surgery in the first Affiliated Hospital of Xi’an Jiaotong University from Jan 2009 to Dec 2020. Included criteria: 1) age 30 to 80 years old; 2) BMI ≥ 18.5 kg/m2; 3) GBC was verified by pathology; 4) underwent radical cholecystectomy in our hospital. 1259 patients were excluded because: 1) without undergone cholecystectomy and verified by pathology; 2) complicated with metastasis of other organs; 3) lack of any of the data. Then 3808 individuals who had undergone health check-ups annually in the same healthcare center were performed a complete clinical and analytical evaluation (including any item following), and choose the newest ones were chosen. Finally, 2210 patients and 2210 health controls aged 30 to 80 years, were chosen after excluding those who: 1) consumed more than 20 g/day of alcohol during the past 12 months (for both men and women) by the diagnostic standard of nonalcoholic fatty liver and the characteristics of Chinese men's drinking habits [12]; 2) BMI < 18.5 kg/m^2^; 3) any missing data including age, gender, height, weight, systolic pressure (SBP), diastolic pressure (DBP), serum total cholesterol (TC), triglyceride (TG), low density lipoprotein (LDL-c), high density lipoprotein (HDL-c), fasting insulin, fasting blood glucose (FBG), retinol binding protein (RBP), or ultrasonographic examination for gallbladder stones and NAFLD; 4) matching failure in any of the following item: age, ethnicity, occupation, and drinking. In additional, we conducted this study in accordance with the Institutional Ethics Committee requirements of the above-mentioned hospital (XJTU1AF2020LSK-160).

### Determination of anthropometric and laboratory parameters

All study points of each GBC patients have been completed by his surgeon in charge during their stay in hospital and stored in our hospital information system. The control participants individually have completed a questionnaire and a measuring scale containing our study items which were stored in our medical information management system.

FBG was determined by the GOD/POD method; Lipid parameters: TC by the CHO/POD method, HDL-C by the AB-Wako method, TG by the GPO/POD method in the blood from the antecubital vein serum were determined in the 10-h fasting state (DiaSys, Holzheim, Germany, all). LDL-C was estimated by the Friedewald equation. Blood RBP and FINS was assayed by immunoturbidimetry or radioimmunoassay, respectively.

A color Doppler ultrasonography instrument (Toshiba, SSA-510A, Japan) was used to identify gallbladder and hepatic diseases. The subjects were fasting and in the supine position. The liver, gallbladder, pancreas and spleen were sequentially examined. GSD and NAFLD were diagnosed as previously reported [12].

### Determination of diabetes, hypertension, hypertriglyceridemia and insulin resistance

Diabetes diagnosis was completed by a professional endocrinologist according to the latest China diagnostic criteria: for example, the newest diabetes patients were diagnosed by Guideline for prevention and treatment of type 2 diabetes mellitus in China 2020 [13]. Hypertension was diagnosed by cardiovascular specialist based on guidelines for pharmacological treatment of hypertension in adults published by the World Health Organization (WHO), for example, SBP ≥ 140 mmHg or DBP ≥ 90 mmHg were diagnosed as hypertension in the latest edition [14]. Homeostasis model assessment-insulin resistance (HOMA-IR) was calculated from fasting glucose and insulin. $HOMA-IR=[FBG\left(mmol/L\right)\times FIN\left(mU/L\right)]/22.5$ [15]. TG > 1.7 mmol/L is defined as hypertriglyceridemia according to the 2018 American Humane Association/American College of Cardiology (AHA/ACC) Guideline [16].

### Statistical analysis

All the data were statistically analyzed with SPSS version 19.0 software (SPSS Inc., Chicago, IL, USA) and the significance level was set at *P* < 0.05. Data are presented as the means ± standard deviations (SD) as the data was normally distributed. To compare variables between two groups or subgroups, Chi-square tests for categorical variables and Student’s t-test for continuous variables were used. We did our mainly analyzed the statistical significance between gender, each of the metabolic syndrome factors, and GBC hazards using multivariate logistic regression model in groups stratified by GBD.

## Results

### General characteristics and serum indexes in the GBC and control groups

The different characteristics in general characteristics and serum indexes between gallbladder cancer and the control group are summarized in Table 1. There were 2210 of 2265 patients with GBC and 2210 subjects of 3808 healthy controls aged 30 to 80 including 1421 (64.3%) and 1034 (46.8%) female participants in GBC and the control group respectively. There was a higher risk of GBC in Female (HR = 2.048, 95%CI: 1.816 ~ 2.311). There were significantly higher BMI, FINS, TG, LDL-c, and HOMA-IR levels and lower SBP, DBP, HDL-c and FBG levels in GSD group versus the controls, and there were the significantly higher incidences of DM, NAFLD and GSD, lower incidence of HBP (*P* < 0.05, respectively), see Table 1.

**Table 1:** The difference of demographic and clinical data of the patients with GBC

	Control group (*N *= 2210)	GBC Group (*N* = 2210)	t or χ^2^	OR	*P* value	95% CI
Female (%)	1034 (46.8)	1421 (64.3)	137.224	2.048	< 0.001	1.816 ~ 2.311
Age (Years)	61.4 ± 11.2	61.4 ± 10.9	0.031	/	0.976	-0.65603 ~ 0.67688
Han (%)	1938 (87.7)	1905 (86.2)	2.172	0.877	0.141	0.736 ~ 1.045
Occupation (%)			1.963*		0.999	
Worker/peasant	608 (27.5)	609 (27.6)	0.001*	1.002	0.973	0.878 ~ 1.144
Professional technician	464 (21.0)	453 (20.5)	0.166*	0.970	0.683	0.839 ~ 1.122
Manager	305 (13.8)	307 (13.9)	0.008*	1.008	0.931	0.849 ~ 1.195
Retiree	700 (31.7)	686 (31.0)	0.206*	0.971	0.650	0.855 ~ 1.103
Others	133 (6.0)	155 (7.0)	1.799*	1.178	0.180	0.927 ~ 1.497
SBP (mmHg)	124.28 ± 17.9	120.24 ± 17.7	-7.380		< 0.001	-5.11896 ~ -2.97014
DBP (mmHg)	77.44 ± 10.5	76.45 ± 10.2	-3.118	/	0.002	-1.61492 ~ -0.36802
FINS (mU/L)	7.86 ± 2.2	15.52 ± 5.9	55.929	/	< 0.001	7.38315 ~ 7.91966
FBG (mmol/L)	5.69 ± 1.4	5.42 ± 1.7	-5.593	/	< 0.001	-0.36593 ~ -0.17598
HOMA-IR	1.95 ± 0.7	3.83 ± 2.3	35.399	/	< 0.001	1.7708 ~ 1.97847
Height (cm)	164.55 ± 8.2	162.66 ± 7.4	-7.883	/	< 0.001	-2.35984 ~ -1.41978
Weight (kg)	65.48 ± 10.6	64.84 ± 17.1	-1.462	/	0.144	-1.49772 ~ 0.21810
BMI (kg/m^2^)	24.12 ± 3.0	24.45 ± 6.0	2.318	/	0.021	0.05195 ~ 0.62199
TC (mmol/L)	4.98 ± 1.0	4.98 ± 1.2	-0.04	/	0.968	-0.06607 ~ 0.06340
TG (mmol/L)	1.55 ± 0.8	2.73 ± 1.4	**33.818**	/	< 0.001	1.10970 ~ 1.24630
HDL-c (mmol/L)	2.97 ± 1.0	2.52 ± 1.1	-14.138	/	< 0.001	0.38842 ~ 0.51349
LDL-c (mmol/L)	2.13 ± 1.4	3.60 ± 1.5	32.606	/	< 0.001	1.38411 ~ 1.56120
Drink (%)	218 (9.9)	207 (9.4)	0.315*	0.944	0.575	0.773 ~ 1.153
HBP (%)	626 (28.3)	431 (19.5)	47.495*	0.613	< 0.001	0.533 ~ 0.705
DM (%)	223 (10.1)	272 (12.3)	5.470*	1.251	0.019	1.036 ~ 1.509
NAFLD (%)	583 (26.4)	811 (36.7)	54.655*	1.618	< 0.001	1.423 ~ 1.839
HTG (%)	546 (25.8)	1852 (87.7)	1791.004*	20.562	< 0.001	17.475 ~ 24.194

*GBC* Gallbladder cancer, *BMI* Body mass index, *TG* Triglyceride, *TC* Total cholesterol, *HDL-c* High density lipoprotein cholesterol, *LDL-c* Low density lipoprotein cholesterol, *NAFLD* Nonalcoholic fatty liver disease, *SBP* Systolic blood pressure, *DBP* Diastolic blood pressure, *FBG* Fasting blood glucose, *FINS* Fasting blood insulin, *HOMA-IR* Homeostasis Model Assessment of insulin resistance, *HBP* high blood pressure, *DM* Diabetes mellitus

^*^mean χ^2^ values for making a difference with t values

### The effects of HTG on GBC

As shown in Table 2, the effects of HTG and GSD on hazard rate of GBC were determined. Significantly higher morbidity with GBC was found in HTG subjects than non-HTG ones in GSD (χ2 = 334.347, OR = 19.077, *P* < 0.001). Similar result was observed in non-GSD (χ2 = 1213.294, OR = 19.906, *P* < 0.001). And Table 1 revealed that HTG also had a higher GBC risk in total (χ2 = 1791.004, OR = 20.562, *P* < 0.001).

**Table 2:** The difference of GBC between HTG and non-HTG

	GSD		Non-GSD	
	The GBC	The control	The GBC	The control
HTG	632*	85	1220^&^	461
Non-HTG	76**	195	182^&&^	1369
Total	708	280	1402	1830

*HTG* Hypertriglyceridemia, *GBC* Gallbladder cancer, *GSD* Gallbladder stone disease

^*^ χ^2^ = 334.347, *P* < 0.001, OR = 19.077, 95% CI: 13.463 ~ 27.034

^&^ χ^2^ = 1213.294, *P* < 0.001, OR = 19.906, 95% CI: 16.492 ~ 24.027

### Relationships between the indexes of BMI, hyperlipidemia, NAFLD and hazard rate of GBC

Table 3 shows the risk factors for developing GBC stratified by GSD based on univariate and multivariate logistic regression. Univariate logistic regression showed significantly positive correlation between female being, hypertriglyceridemia, serum TG, LDL-c levels, HOMA-IR, FINS, and hazard of GBC (*P* < 0.05, all), while significantly negative correlation between serum HDL-c, FBG levels, SBP, DBP, HBP and hazard of GBC (*P* < 0.05). Of them, serum TG level was the most remarkable hazardous factor of GBC with GSD (OR = 37.784, *P* < 0.001). Multivariate logistic regression without HTG showed that there was significantly positive correlation between, HOMA-IR, serum TG, LDL-c and hazard of GBC (*P* < 0.05, all), while significantly negative correlation between serum HDL-c levels, HBP and hazard of GBC (*P* < 0.001, all). Of them, serum TG was the the most significantly positive association to GBC (OR = 16.912, *P* < 0.001). Similar results were observed by multivariate analysis including HTG. Of them, HTG was the most significantly positive association to GBC (OR = 10.636, *P* < 0.001).

**Table 3:** Univariate and multivariate analyses of gallbladder cancer risk in GSD patients

Factors		Univariate				Multivariate (without HTG)			Multivariate (with HTG)			
	β	OR	95%CI	*P* value	β	OR	95%CI	*P* value	β	OR	95%CI	*P* value
Gender (F)	0.877	2.404	1.796 ~ 3.218	< 0.001	0.487	1.627	0.954 ~ 2.776	0.074	0.586	1.796	1.110 ~ 2.908	0.017
BMI	-0.009	0.991	0.953 ~ 1.031	0.662	-	-	-	-	-	-	-	-
TG	**3.549**	**34.784**	**20.961 ~ 57.723**	**< 0.001**	**2.828**	**16.912**	**9.648 ~ 29.643**	**< 0.001**	/	/	/	/
TC	-0.097	0.908	0.816 ~ 1.010	0.076	-	-	-	-	-	-	-	-
HDL-c	-0.326	0.722	0.633 ~ 0.823	< 0.001	-0.481	0.618	0.486 ~ 0.787	< 0.001	-0.407	0.665	0.537 ~ 0.824	< 0.001
LDL-c	1.845	6.331	4.894 ~ 8.189	< 0.001	1.205	3.338	2.476 ~ 4.498	< 0.001	1.442	4.230	3.172 ~ 5.641	< 0.001
HOMA-IR	1.148	3.153	2.587 ~ 3.842	< 0.001	0.968	2.633	2.022 ~ 3.428	< 0.001	0.878	2.407	1.892 ~ 3.063	< 0.001
DM	0.030	1.030	0.686 ~ 1.548	0.886	-	-	-	-	-	-	-	-
NAFLD	0.017	1.017	0.766 ~ 1.352	0.905	-	-	-	-	-	-	-	-
DBP	-0.054	0.947	0.934 ~ 0.960	< 0.001	/	/	/	/	/	/	/	/
SBP	-0.027	0.974	0.966 ~ 0.981	< 0.001	/	/	/	/	/	/	/	/
FINS	0.427	1.533	1.441 ~ 1.631	< 0.001	*	*	*	*	*	*	*	*
FBG	-0.079	0.924	0.860 ~ 0.994	0.034	*	*	*	*	*	*	*	*
HBP	-0.811	0.445	0.330 ~ 0.598	< 0.001	-0.934	0.393	0.226 ~ 0.682	0.001	-0.737	0.479	0.292 ~ 0.784	0.003
HTG	2.949	19.077	13.463 ~ 27.034	< 0.001	/	/	/	/	2.364	10.636	6.644 ~ 17.028	< 0.001

*BMI* Body mass index, *TG* Triglyceride, *TC* Total cholesterol, *HDL-c* High density lipoprotein cholesterol, *LDL-c* Low density lipoprotein cholesterol, *NAFLD* Nonalcoholic fatty liver disease, *SBP* Systolic blood pressure, *DBP* Diastolic blood pressure, *FBG* Fasting blood glucose, *FINS* Fasting blood insulin, *HOMA-IR* Homeostasis model assessment of insulin resistance, *HBP* High blood pressure, *DM* Diabetes mellitus

^*^eliminated for the definition of HOMA-IR in multivariate regression

— eliminated for no statistical significance in univariate regression

/ eliminated for the collinear/redundant correlation between SBP, DBP and HP; TG and HTG in multivariate regression

To investigate the possible reason that caused above result, we further analyzed data stratified by non-GSD based on univariate and multivariate logistic regression analysis (as Shown in Table 4). Univariate logistic regression showed significantly positive correlation between female being, BMI, DM, HTG, NAFLD, serum TG, LDL-c levels, HOMA-IR, FINS and hazard of GBC (*P* < 0.05, all), and significantly negative correlation between serum HDL-c, FBG levels, SBP, HBP and hazard of GBC (*P* < 0.05). Multivariate logistic regression showed that there was significantly positive correlation between female being, serum TG, LDL-c, HOMA-IR levels and hazard of GBC (*P* < 0.05, all), and significantly negative correlation between serum HDL-c levels, DM, HBP and hazard of GBC (*P* < 0.05, all). Of them, HOMA-IR was the most significantly positive association to GBC with (OR = 8.739, *P* < 0.001).

**Table 4:** Univariate and multivariate analysis of gallbladder cancer risk in the patients without GSD

	Univariate				Multivariate			
	β	OR	95%CI	*P* value	β	OR	95%CI	*P* value
Gender(F)	0.627	1.872	1.624 ~ 2.157	< 0.001	0.671	1.956	1.502 ~ 2.546	< 0.001
BMI	0.031	1.032	1.010 ~ 1.055	0.004	0.014	1.014	0.993 ~ 1.035	0.184
TG	1.739	5.690	4.985 ~ 6.494	< 0.001	1.284	3.611	3.056 ~ 4.267	< 0.001
TC	0.030	1.030	0.962 ~ 1.104	0.398	-	-	-	-
HDL-c	-0.433	0.649	0.605 ~ 0.696	< 0.001	-0.534	0.586	0.516 ~ 0.666	< 0.001
LDL-c	1.124	3.078	2.810 ~ 3.372	< 0.001	0.889	2.432	2.147 ~ 2.756	< 0.001
HOMA-IR	1.695	5.447	4.791 ~ 6.192	< 0.001	**2.168**	**8.739**	**7.092 ~ 10.769**	** < 0.001**
DM	0.238	1.269	1.019 ~ 1.579	0.033	-4.133	0.016	0.008 ~ 0.033	< 0.001
NAFLD	0.565	1.760	1.515 ~ 2.045	< 0.001	-0.305	0.737	0.506 ~ 1.074	0.113
DBP	-0.004	0.996	0.990 ~ 1.003	0.309	-	-	-	-
SBP	-0.011	0.989	0.985 ~ 0.993	< 0.001	**/**	**/**	**/**	**/**
FINS	0.571	1.771	1.701 ~ 1.843	< 0.001	*	*	*	*
FBG	-0.135	0.874	0.830 ~ 0.920	< 0.001	*	*	*	*
HBP	-0.491	0.612	0.518 ~ 0.723	< 0.001	-0.430	0.650	0.456 ~ 0.928	0.018
HTG	2.991	19.906	16.492 ~ 24.027	< 0.001	**/**	**/**	**/**	**/**

*BMI* Body mass index, *TG* Triglyceride, *TC* Total cholesterol, *HDL-c* High density lipoprotein cholesterol, *LDL-c* Low density lipoprotein cholesterol, *NAFLD* Nonalcoholic fatty liver disease, *SBP* Systolic blood pressure, *DBP* Diastolic blood pressure, *FBG* Fasting blood glucose, *FINS* Fasting blood insulin, *HOMA-IR* Homeostasis Model Assessment of insulin resistance, *HTG* Hypertriglyceridemia, *HBP* High blood pressure, *DM* Diabetes mellitus

^* ^eliminated for the definition of HOMA-IR in multivariate regression

— eliminated for no statistical significance in univariate regression

/ eliminated for the collinear/redundant correlation between SBP and HBP; TG and HTG in multivariate regression

## Discussion

Epidemiological studies have identified that GSD is one of several GBC risk factors, despite epidemiologic data, definitive evidence for the role of gallstones as a cause for gallbladder cancer is lacking, GSD might associate to the elevated hazard of GBC by 2.4-fold (gallstone diameter = 2.0 ~ 2.9 cm) to 9.2–10.1-fold (gallstone > 3 cm) [17, 18]. In this clinical case–control study matched for ethnicity, occupation, and drinking conducted in northwestern China, we identified that Female, insulin resistance and hypercholesterolemia were independent risk factor of GBC predicting [8, 19]; and DM didn’t associate significantly with GBC in GSD patients as the recent report [20]. Interestingly, our study revealed initially that TG was the most remarkable independent predicting factor of GBC risk in GSD patients among metabolic/environmental factors; while HTG was in the patients without GSD.

First, our study showed that DM was not significantly correlated to GBC risk in the GSD patients, which HOMA-IR was a significant position to GBC risk in the patient without or with GSD. We tend to consider hyperinsulinemia as an independent predicting factor of GBC risk [21], while DM as a risk factor for gallbladder cancer as a result of synergistic hypertension and hyperlipidemia [20]. The recent study reported that insulin might reduce the levels of insulin growth factor binding proteins (IGFBP) 1 and IGFBP2 in the circulation, resulting in the increase of circulating insulin growth factor (IGF); the latter and insulin might stimulate target cells toward malignant transformation [22]. In additional, few study has clarified that LDL-c is associated with GBC, while it is undoubted that very low dentine lipid (VLDL) receptor has been reported to be involved in the pathogenesis of GBC by regulating the expression of the components of the fibroblast growth factor receptor signaling pathway through Mitogen-Activated Protein Kinase (MAPK) [23], while high LDL-c levels could interrupt "endogenous lipid pathway" as result of VLDL accumulation because HTG will lead to the formation of large triglyceride-rich VLDL particles due to the fact that triglycerides overproduction is disproportionately greater than increasing apoB production, especially as seen in the insulin resistant state [24].

Second, our result revealed that HTG was significant positive correlated to GBC risk in GSD patients, and elevated serum TG was an independent risk factor of GBC predicted as the most remarkable. It might be because: there are little data on the role of elevated TG-related biomarkers on gallbladder cancer risk, but HTG, relating to GSD, might decrease sensitivity to cholecystokinin, and increase both biliary cholesterol saturation and bile viscosity enhancing mucin production [25, 26], and cholesterol stones and gallbladder infections were associated with increased MUC3 and MUC5B expression [27]. Epidemiological data have revealed that gallbladder stone is considered the most important risk factor for GBC, and the large volume or high weight of gallstones could further increase the risk [28]. The synergistic effect of the gallbladder stones and infections leads to the thickening of gallbladder mucosa, which leads to the occurrence of gallbladder cancer in symptomatic GSD patients. Even so, it is known that the synthesis of TG requires glycerol and fatty acids, most of which are for glucose metabolism origin; the former is converted from dihydroxyketone phosphate produced by glycolysis; the latter is synthesized from acetyl CoA produced by oxidative decomposition of glucose. In short, continuous elevated TG might infer to accumulation of glucose or insulin resistant, with which blood glucose accumulation is transformed into TG by hepatocytes and TG is released into blood, resulting in the increase of TG concentration in blood [24]. Several prospective epidemiological studies showed that excess body weight in combination with physical inactivity is a major determinant for the development of insulin resistance with associated with hyperglycemia and hyperinsulinemia and further leads to tumor development by several biological pathways, such as chronic low-grade inflammation, glucose toxicity, advanced glycosylated end product metabolism and the adenosine monophosphate kinase pathway [21]. Clearly, elevated blood TG is the most remarkable independent predicting factor of GBC risk, possibly because elevated TG is associated with GSD [24], and other indexes of metabolic syndrome [29], yet the effect of the latter is possibly interfered constructively or destructively. Speculatively, our result, the observed persistent increase in serum TG, might be considered as the potential of a candidate diagnostic or prognostic biomarker of GBC in the patients with GSD.

Third, our study revealed that HTG is a significant predicting factor of GBC risk including in the patients without GSD, the data revealed that hypertriglyceridemia might increase the risk of prostate cancer [30], hepatocellular cancer [31], endometrial cancer [32], pancreatic cancer [33] and so on, which tended to consider the above correlation between HTG and GBC due to high secretion of bile acids, while bile acid metabolic dysfunction is a causal factor of gallstones [33, 34]. Thus, we speculate that obesity and/or insulin resistance should be the main causes in patients without GSD [35], as our multivariate logistic regression showed that HOMA-IR was the most remarkable independent predicting factor of GBC risk. There is convincing evidence that obesity may disturb lipid and endogenous hormones metabolism, result in gallbladder dysfunction, increase the risk of gallstones, and thus play a role in GBC [9, 36], while the recent study supported that obesity might be protective factor for several cancers (for example premenopausal breast cancer, non-small cell lung cancer and head and neck cancers) [35]. Undoubtedly, central accumulation of body fat is associated with insulin resistance [37], which has a role in the aetiology of biliary tract cancers (including GBC) [38]; insulin could stimulate the insulin receptor or the IGF-1R, activate their intrinsic tyrosine kinase activity, induce the production of lipid messengers by the phosphatidylinositol 3-kinase, and then trigger AKT-mammalian target of rapamycin pathway regulating cell growth and differentiation and the Ras-Raf-MEK-MAPK pathway inducing cell proliferation [39]. Moreover, insulin might upregulate the metabolic activity of the cell, producing reactive oxygen species causing DNA double strand break or mutation in vitro [40].

### Strengths and limitations

We reviewed a large number of participants’ case history with more than ten years, making sure that the patients had been diagnosed by surgery and pathology in the case group and the matched participants come from the same occupation, and similar environment. However, the present study has several limitations. First, this is not a multicenter and cohort study, and statistics may be biased. Secondly, we provided little enlightenment about the cause for the negative correlation between HBP, SBP, DBP and GBC, even HBP might be an independent protective factor in our study. Maybe elevated neuropeptides vasoactive intestinal peptide levels linking gastrointestinal motility regulation reduce diarrhea, secondary to avoiding HTG, which needs the further proof of animal and molecular epidemiological study [41]. Third, there might be errors in the family history of cancer due to the small family units in China and Chinese personality characteristics; therefore, we haven’t completed an accurate data on family history of cancer and matching, as these statistics may be biased. Final, in the exclusion criteria of nonalcoholic fatty liver disease, we chose the same threshold for men and women, which may have a certain import on the results.

## Conclusions

In summary, we found that hyperlipidemia, insulin resistance, Female may be associated with GBC risk in GSD patients. Of them, elevated serum triglyceride levels could be the remarkable independent predicting factor of GBC risk. More importantly, it is initially found that the sharp rise of serum TG levels might be advocated as the potential of a candidate diagnostic or prognostic biomarker of GBC with GSD. Meanwhile, we clarified that insulin resistance is a remarkable independent predicting factor of GBC risk without GSD. However, the viewpoint needs to be confirmed in the next step multicenter, cohort studies.

## Data Availability

Not applicable. The authors declare that the data supporting the findings of this study are available within the article.

## Abbreviations

GBC: Gallbladder cancer
BMI: Body mass index
TG: Triglyceride
TC: Total cholesterol
HDL-c: High density lipoprotein cholesterol
LDL-c: Low density lipoprotein cholesterol
NAFLD: Nonalcoholic fatty liver disease
SBP: Systolic blood pressure
DBP: Diastolic blood pressure
FBG: Fasting blood glucose
FINS: Fasting blood insulin
RBP: Retinol binding protein
HOMA-IR: Homeostasis Model Assessment of IR
IR: Insulin resistance
HBP: High blood pressure
DM: Diabetes mellitus
MetS: Metabolic syndrome
IGF: Insulin growth factor
NASH: Non-alcoholic steatohepatitis
HCC: Hepatocellular carcinoma
VLDL: Very low-density lipoprotein
IGFBP: Insulin growth factor binding proteins
MAPK: Mitogen-Activated Protein Kinase
